# Full Factorial Design for Gold Recovery from Industrial Solutions

**DOI:** 10.3390/toxics9050111

**Published:** 2021-05-20

**Authors:** Maria Mihăilescu, Adina Negrea, Mihaela Ciopec, Petru Negrea, Narcis Duțeanu, Ion Grozav, Paula Svera, Cosmin Vancea, Alina Bărbulescu, Cristian Ștefan Dumitriu

**Affiliations:** 1 Research Institute for Renewable Energies, Politehnica University Timişoara, 2, P-ța Victoriei, 300006 Timişoara, Romania; maria.mihailescu@upt.ro; 2 Faculty of Industrial Chemistry and Environmental Enginering, Politehnica University Timişoara, 2, P-ța Victoriei, 300006 Timişoara, Romania; adina.negrea@upt.ro (A.N.); mihaela.ciopec@upt.ro (M.C.); petru.negre@upt.ro (P.N.); narcis.duteanu@upt.ro (N.D.); ion.grozav@upt.ro (I.G.); cosmin.vancea@upt.ro (C.V.); 3 National Institute for Research-Development for Electrochemistry and Condensate Matter, 114, Dr. A. Păunescu Podeanu Str., 300224 Timişoara, Romania; paula.svera@upt.ro; 4 Department of Civil Engineering, Transilvania University of Brașov, 5 Turnului Str., 900152 Brașov, Romania; 5 SC Utilnavorep SA, Constanța, 55, Aurel Vlaicu Bd, 90055 Constanța, Romania

**Keywords:** Amberlite XAD7, Am-L-GA, L-glutamic acid, factorial design

## Abstract

Gold is one of the precious metals with multiple uses, whose deposits are much smaller than the global production needs. Therefore, extracting maximum gold quantities from industrial diluted solutions is a must. Am-L-GA is a new material, obtained by an Amberlite XAD7-type commercial resin, functionalized through saturation with L-glutamic acid, whose adsorption capacity has been proved to be higher than those of other materials utilized for gold adsorption. In this context, this article presents the results of a factorial design experiment for optimizing the gold recovery from residual solutions resulting from the electronics industry using Am-L-GA. Firstly, the material was characterized using atomic force microscopy (AFM), to emphasize the material’s characteristics, essential for the adsorption quality. Then, the study showed that among the parameters taken into account in the analysis (pH, temperature, initial gold concentration, and contact time), the initial gold concentration in the solution plays a determinant role in the removal process and the contact time has a slightly positive effect, whereas the pH and temperature do not influence the adsorption capacity. The maximum adsorption capacity of 29.27 mg/L was obtained by optimizing the adsorption process, with the control factors having the following values: contact time ~106 min, initial Au(III) concentration of ~164 mg/L, pH = 4, and temperature of 25 °C. It is highlighted that the factorial design method is an excellent instrument to determine the effects of different factors influencing the adsorption process. The method can be applied for any adsorption process if it is necessary to reduce the number of experiments, to diminish the resources or time consumption, or for expanding the investigation domain above the experimental limits.

## 1. Introduction

Precious metals from the platinum group (PMs) have various applications in different fields due to their physical and chemical properties, such as catalytic activity, good electrical conductivity, corrosion resistance, etc. [1,2]. Over the last 40 years, considerable amounts of gold have been used in the electronics industry [3,4]. Gold is utilized in the technique of thin starts, printed circuit boards manufacturing, for high-frequency conductors, shielding and surface protection, contact mechanism (contact with plugs), and for connecting surfaces in cable structures of electronic assemblies [5]. Its value, rarity, exceptional properties, and diverse utilizations (due to the resistance to corrosion, good electrical conductivity, and high catalytic activity) increased the demand for gold, making its recovery very important [6].

From an environmental viewpoint, for some metals, scientists estimated [7,8] that nowadays extracting the same amount as a century ago necessitates the removal of about three times more rocks. Therefore, recycling is beneficial from economic and environmental perspectives. Recycling also is a valuable source to recover scarce materials [7,9,10,11].

Even if gold is not generally considered a dangerous metal for human health, recent studies showed the adverse effects of gold on human health [12,13,14,15]. It was proved that metallic gold induces allergic contact hypersensitivity [12]. Furthermore, by gold ingestion, the blood enzymes decrease, suggesting the inhibition of enzymatic activity. A decrement in the serum enzymes (CPK and LDH) was also noticed [13]. Experiments on mice found that the intermediate gold nanoparticles with dimensions of 8–37 nm produced severe sickness, loss of appetite, weight loss, change in fur color, and shorter average lifespan (the majority died within 21 days), while systematic toxicity of gold particles with the range 18–37 nm was linked to major damage of the liver, spleen, and lungs [14]. The effects of the dimensions and surface capping of nanogold have been systematically studied [15]. Since many aspects of the effects of gold toxicity on human health are still unknown, it is better to prevent its ingestion and recover it from the waste solutions that could pollute the environment.

For these above reasons, gold recovery is very important. Large-scale processes, such as mechanical separation, pyro-metallurgy, hydrometallurgy, or bio-hydrometallurgical technologies, are currently used for this purpose [4,16,17,18,19].

The most common method employed for recovering gold, a cyanide-leaching agent, is toxic. To solve this problem, the following elements are utilized as cementing agents: magnesium [20], copper [20], iron [21], zinc [22], or aluminum [23]. The reduction process uses hydrazine [24] or sodium borohydride [25], or ferrous sulfate, sodium sulfite, potassium sulfite, and sulfur dioxide [26].

Due to the low concentrations of gold in waste, refining processes, such as chemical treatments (chemical dissolution, reduction, etc.), adsorption on resins followed by incineration are required [27]. The most widely utilized adsorbent material was activated carbon [28]. Over time, new materials, such as thiourea [29], or natural materials, like barley, apricot shells, or rice, have been used in gold retrieval from waste solutions [30]. The silica’s chemical functionalization with hanging groups resulted in new materials that improved the adsorbent properties [31].

The control of the adsorption’s process-specific parameters/characteristics is the key to a maximum adsorption capacity. Therefore, it is essential to identify the parameters that predominantly affect the adsorption performance. In some cases, the effect of a specific parameter on the adsorption capacity may differ when other control parameters are modified, leading to interaction effects. The conventional single variable approach can require a large number of experiments to find the optimal conditions. Through the experiments’ factorial design, multiple control parameters are systematically varied to study their effects on adsorption (the response variable) [32,33,34]. A significant advantage of the factorial design is the accurate estimation of each factor’s main effects [35].

Given its advantages, this method has been used for optimizing different extraction processes [36,37,38,39,40]. Zhao et al. [36] used the factorial design for the study of phosphate removal from aqueous solutions; Shah and Garg [37] applied a 2^k^ full factorial design in the optimization of solvent-free microwave extraction of ginger essential oil; Kumar et al. [38] validated the use of HPLC to determine valsartan in nanoparticles; and Gabor et al. [39] employed the method for optimizing the lanthanum adsorption process onto chemically modified biomaterials. Only recently have a few studies [41,42,43] relied on a factorial design to optimize gold recovery. Mendes and Martins [41] and Oluwabunmi et al. analyzed the extraction from gold and gold–copper ores, respectively, while Teimouri et al. [42] reported the refractory sulphidic gold tailings with ionic liquids.

In this context, the goal of this research was to optimize the gold extraction from industrial waste diluted solutions using a new adsorbent material, synthesized by us [44], which is a commercial Amberlite XAD-type resin functionalized by chemical modification with L-glutamic acid (Am-L-GA), using the dry solvent-impregnated resin (SIR) method. At the first stage, the material’s morphology was analyzed by AFM to emphasize the roughness parameters, which are important for the adsorption capacity. Then, a two-level factorial design experiment was performed to investigate the influence of four factors (pH, contact time, temperature, and initial concentration) on the material adsorption capacity. It is emphasized that a factorial design method is an appropriate tool to investigate the effects of many factors on the adsorption capacity of Am-L-GA, thus reducing the number of experiments. It can be successfully used not only for optimizing the gold recovery process by adsorption but also for other similar processes.

## 2. Materials and Methods

Residual solutions resulting from the electronics industry, from the industrial process of gold-plating of copper coatings on printed circuit boards, were used in this study. They contain K[Au(CN)_2_], cyanide complexes of Au (III), KCN, Ni, and Cu (II).

The electrodeposition based on the double salt gold potassium cyanide is not a new procedure [45]. Pure gold electroplated from neutral or alkaline electrolytes cannot be utilized for applications where contacts and connectors are employed because of its thermo-compression ability. The pure gold deposit is capable of welding to itself when contact is made, resulting in a permanently closed contact. Therefore, gold is co-deposited with a transition metal—Ni, Cu, or Fe—leading to an increase in the deposit hardness and wear resistance. The presence of these impurities reduces the tendency of the gold layer to weld by friction, making the material suitable for connectors, sliding contacts, and contact applications. The typical bath composition and operating conditions are presented in [45], whereas the deposition reactions are presented in [46]. We do not insist on this procedure, since our goal is not the gold deposition study, but the gold removal from the residual solution resulting after this procedure. For a deeper insight, the reader may see [45,46,47,48].

The residual solutions resulting after the gold-plating procedure utilized in our research contained 2 g/L gold ions, 0.7–1 g/L Ni (II), 1 mg/L Cu (II), and 2 mg/L Fe (III), which were determined in these solutions before the experiments [44].

### 2.1. Material Preparation

To determine the equilibrium concentration and the effect of the initial concentration of Au (III) on the material adsorption capacity, the following procedure was performed. A residual solution of an industrial cyanide bath was treated with HCl (37 wt.%, Sigma Aldrich) and HNO_3_ 63.013 wt.%, Chem Spider) to obtain a solution containing 2 g Au (III)/L. Further, this stock solution was diluted to prepare a solution of 100 mg Au (III)/L; by further dilution, Au (III) solutions of different concentrations (5, 10, 25, 50, 75, and 100) were also prepared.

The adsorbent material Am-L-GA was obtained from 0.1 g of L-glutamic acid (puriss. >99.0%, Merck, dissolved in 25 mL distilled water acidified with 37% HCl (Merck). The dissolved extractant was mixed with 1 g of Amberlite solid support XAD7 (20–60 mesh, Sigma-Aldrich, Merck), considering a solid-to-extractant ratio of 10:1. Functionalization was performed by keeping the support and the extractant in contact for 24 h, then dried in an oven (Pol—eko model SLW 53, SDT Poland) for 24 h at 50 °C.

The L-Glutamic acid content of the impregnated resin, Amberlite XAD 7, was determined after washing with H_2_O (which completely eluted the extractant) followed by subsequent titration with 0.1 M NaOH [49]. The extractant content of the L-Glutamic acid impregnated XAD-7 material was 0.32 g L-Glutamic acid impregnated resin [44,50]. Notice that in this procedure adsorption is mainly a physical process.

The initial laboratory adsorption study was performed by varying the temperatures in the range 25–45 °C, the contact time in the range of 15–120 min, pH in the range of 2–14, and the initial concentrations in the range 5–100 mg Au (III)/L. The experiments were carried out in triplicate, with standard deviations of the obtained values under 1%. In previous studies [44,50], the authors revealed more details of the experiments.

### 2.2. Material Characterizing

The obtained material (Am-L-GA) was characterized using atomic force microscopy (AFM). AFM images were obtained by the Scanning Probe Microscopy Platform (MultiView-2000 system, Nanonics Imaging Ltd., Jerusalem, Israel) using only the intermittent mode in normal conditions (298 K). The analysis used a chromium-doped tip with a 20-nm radius and 30–40 kHz resonance. The AFM analysis was conducted to observe the sample morphology. The SEM analyses are reported in [44,50]. AFM can provide additional details regarding the roughness of the material’s surface that the SEM method is unable to. The roughness data is important for evaluating the material properties, especially the capacity of adsorption on its material [51]. Details are provided in the section Results and Discussion.

### 2.3. Factorial Design Experiments

The statistical methods represent an advanced tool helping researchers to better understand and optimize controllable process variables, playing an important role in planning, analyzing, and interpreting data resulting from experimental determinations, which allows optimal management of industrial processes.

**Figure 1:**
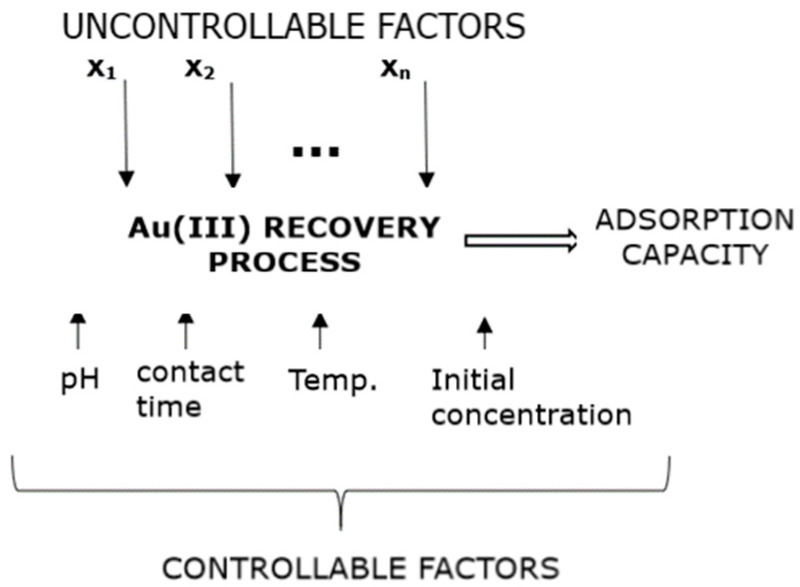
Adsorption process model.

The factorial design is one of the most common methods for studying each factor’s effect and their interactions on the dependent variable. In this case, the response variable is the adsorption capacity, and the factors are the pH of the metal ion solution, the contact time, the solutions’ temperature, and the initial concentration of the metal ion in the solution. Figure 1 presents a suggestive illustration of the studied adsorption model.

Suppose that multiple variables influence the adsorption process and, implicitly, the adsorption capacity of the studied material. In that case, it is indicated to design the experiment using statistical systems so that when the experiment ends, there is the possibility of choosing reliable and economically efficient solutions [32,35,36].

The purpose of this factorial design study is to optimize the recovery process of Au (III) from diluted industrial solutions by adsorption on the Am-L-GA material. The aim is to establish the parameters that significantly influence the adsorption capacity (*q*) and optimize the controllable factors’ values (pH, contact time, temperature, and initial concentration) for conducting the adsorption process to obtain the desired results. Therefore, the experiment was carried out in two phases. In Phase 1, the objectives were establishing the variables that have an essential role in the adsorption process and determining the controllable factors to obtain the maximum adsorption capacity. In Phase 2, the goal was to design the response surface models and optimize the adsorption process for obtaining the maximum value of the adsorption capacity. Thus, the essential controllable factors that lead to the maximum value of the adsorption capacity were established using the response surface models [39].

The variables’ values were taken from the experimental results [44,50], but some ranges were extended as follows. The pH and temperature values were kept as in the experiments. The gold’s initial concentrations were considered in the range 5–175 mg Au (III)/L, and the contact time between 15 and 240 min.

## 3. Results and Discussion

### 3.1. Material Characterization by Atomic Force Microscopy (AFM)

The synthesized material was analyzed by X-ray energy dispersion (EDX) using a Quantum FEG 250 scanning electron microscope, and Fourier Transformed Infrared spectroscopy (FTIR) using a Bruker FT-IR spectrometer Platinum ATR-QL Diamond, in the range 4000–400 cm^−1^. The adsorbent material obtained after Amberlite XAD7 functionalization was dried for 24 h; afterward, several granules of modified adsorbent were glued on the carbon adhesive disks and fixed on the stabs. These stabs were further used for data collection [50].

An important factor for the adsorption process of different metal ions is represented by the structure of the adsorbent material. In the present case, for a better understanding of the results obtained by the factorial design, the adsorbent material was first analyzed using the AFM technique.

It is known that the most commonly used methods for surface investigation are Scanning Electron Microscopy (SEM) and Scanning Probe Microscopy (SPM). AFM has become the most-used technique of SPM, serving only for the topographic analysis of the surfaces, highlighting the morphological changes due to the chemical functionalization. AFM generates three-dimensional images of the surfaces with a nano-lateral and sub-angstrom-vertical resolution. The great AFM advantage is that it can operate in air, vacuum, and liquids at different temperatures.

The AFM topography has a nanometric resolution in the x and y planes and sub-nanometric resolution in the z direction and can be obtained in two ways: contact and tapping (named according to the manufacturer: AC, semi-contact, intermittent contact). By recording the height at each scan point, a 3D image of the surface is obtained. In the tapping mode, the microcantilever oscillates at a frequency close to its resonant frequency, with the amplitude of the oscillation being monitored. During scanning, the cantilever vibrates so that the AFM needle periodically touches the surface of the sample, avoiding friction between the needle and the surface present in contact mode, preventing the sample’s destruction. When scanned in the x and y planes, 2D images are obtained [52].

As mentioned before, surface morphology plays a significant effect on adsorption. The characterization of materials through their roughness allows one to obtain essential information to achieve material efficiency [53,54]. The origin of the adhesion force is the same as the force nature responsible for the binding of atoms and molecules. The adhesion phenomenon is important in different applications [55], as in the present one.

**Figure 2:**
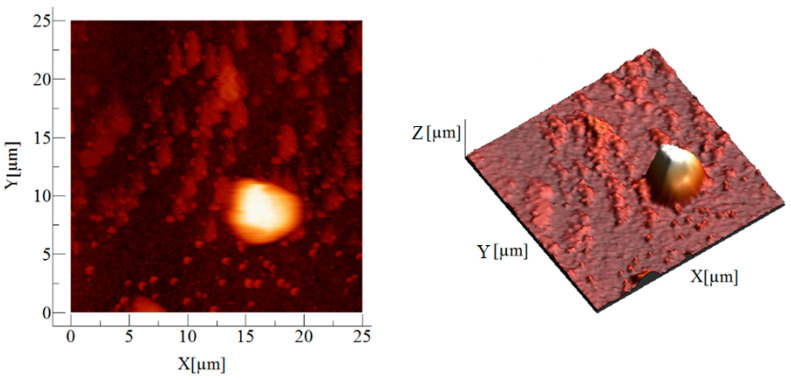
2D and 3D image of the Am-L-GA sample on 25 µm × 25 µm area (Z = 1.9 μm in the 3D representation).

**Figure 3:**
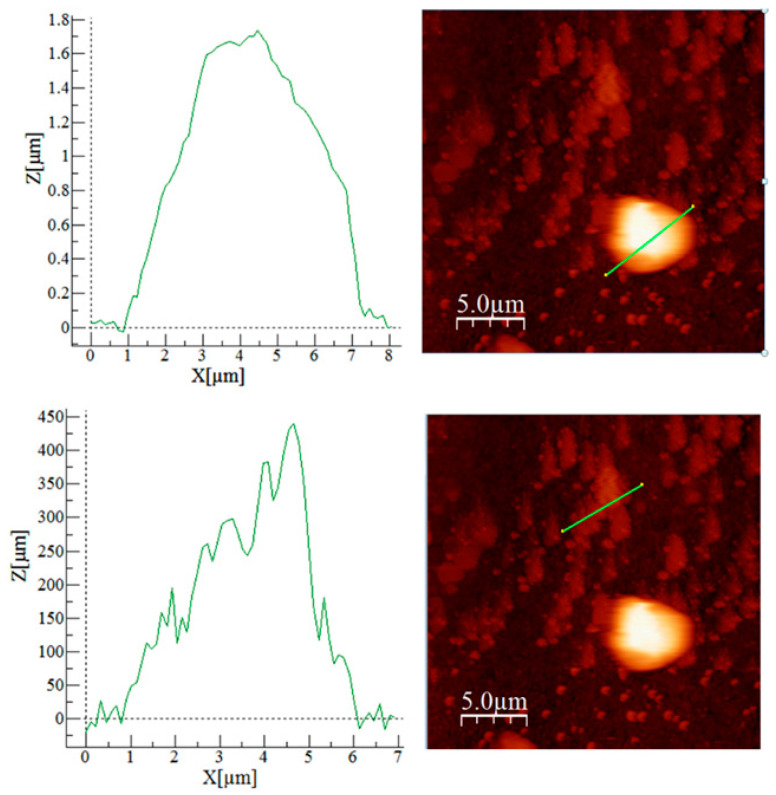
Atomic force microscopy (AFM) image on two selected zones—green lines.

**Figure 4:**
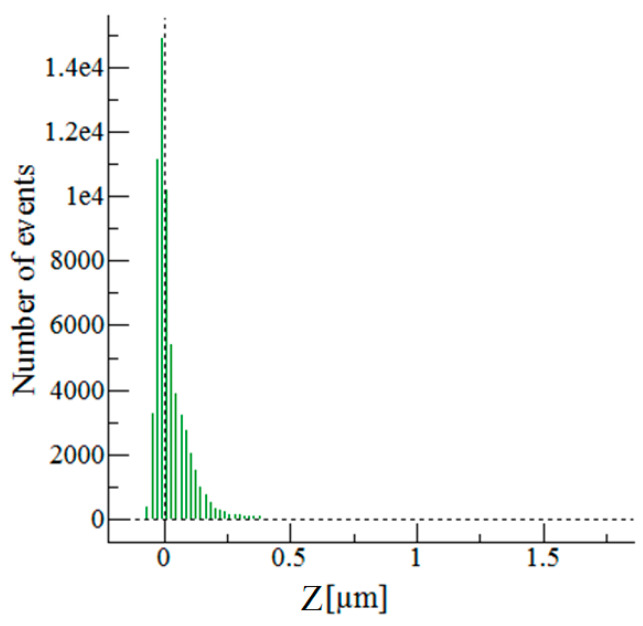
Height distribution of the 25 µm × 25 µm analyzed area.

AFM images for the Am-L-GA material are shown in Figure 2, Figure 3 and Figure 4.

Figure 3 shows the formation of similar morphology patterns (with ≈0.45 μm height) combined with a round-shaped formation (with ≈1.70 μm) that exhibits higher big values (seen in the image with roughness analysis on a selected area, Figure 3).

Figure 4 represents a graph with the most height/valley distribution points on the whole analyzed area, whereas the range between 0.1 and 0.25 µm contains most of the heights/valleys.

The basic statistics values computed from the AFM images (average roughness (Sa), mean square root roughness (Sq), maximum peak height (Sp), maximum valley depth (Sv), maximum peak-to-valley height (Sy), surface kurtosis (Sku), and surface skewness (Ssk)) are presented in Table 1.

Both Sa and Sq are used to evaluate the material’s average roughness, whereas Sa is generally used to display the surface roughness. Sq represents the root mean square value of ordinate values within the definition area [56].

Another parameter, skewness (Ssk), is an indicator of occasional deep valleys or high peaks in the profile due to its ability to measure the symmetry of the variation in a profile on the mean line. In this case, positive skewness (Ssk > 0) was observed, indicating the presence of high spikes that protrude above the flatter average.

Kurtosis (Sku) describes the density sharpness of the profile; more precisely, the presence of both high peaks and low valleys, which are reflected in a kurtosis with a value less than 3, whereas a kurtosis with a value higher than 3 confirms the presence of numerous high peaks and low valleys. The height of the highest peak within the defined area was determined from the Sp value, while the Sv value confirmed the height of the largest pit within the same defined area. Sy is defined as the sum of the largest peak height value (Sp) and the largest pit depth value (Sv) within the defined area [56].

The sample shows the prevalence of peaks (higher Sp value compared to Sv value), resulting in a positive Sy, which is also confirmed by the Ssk value. The Ssk value is also an excellent indicator for the material’s porosity, with a negative skewness (Ssk < 0) pointing out the presence of porous surfaces. The Ssk value for Am-L-GA indicates low porosity, a characteristic that is also visible in the cross-section image in Figure 3. In the same image, a roughness analysis was performed on two distinct areas, demonstrating the material’s different surfaces, which is also visible in the 2D image. Another aspect of the Ssk parameter that can be taken into account is the lower static coefficient of the present materials (high Sku value and positive Ssk value) by comparison to surfaces with a Gaussian distribution (Sku = 3, Ssk = 0). All these parameters indicate an appropriate material structure, recommended for the adsorption process [53,54,55,56].

### 3.2. Fatorial Design Experiments

#### 3.2.1. Phase 1: Linear Experiments

**Figure 5:**
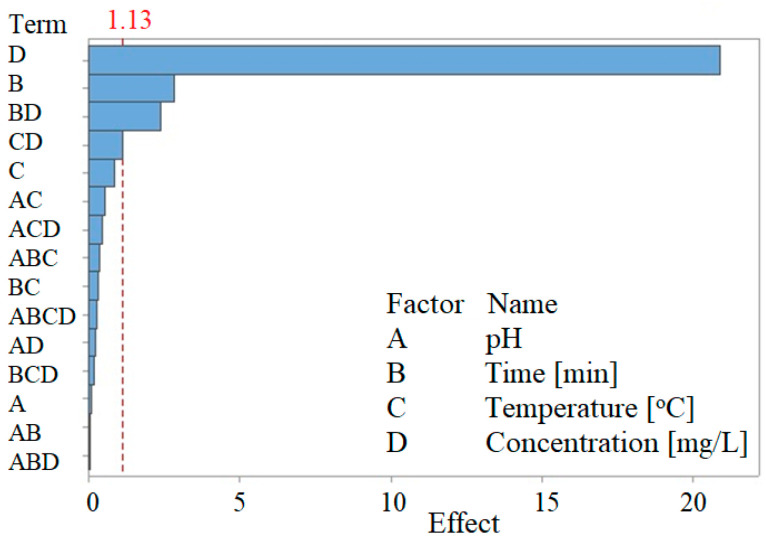
Pareto diagram—effect of the control parameters on adsorption capacity.

In this first stage, a complete factorial design model was developed, having four controllable factors, namely, the pH of the solution, the contact time, the temperature, and the initial concentration of Au (III). Sixteen runs were performed using the statistical software MINITAB 15. The Pareto diagram, which illustrates the effect of the control parameters on the response variable (which is the adsorption capacity), is presented in Figure 5.

The regression equation in non-coded units is

CapAds = −1.207 + 0.06598 A + 0.009908 B + 0.03695 C + 0.1081 D − 0.001356 AB – – 0.005016 AC+ 0.01437 AD − 0.000204 BC + 0.000624 BD + 0.000332 CD + 0.000039 ABC– – 0.000091 ABD − 0.000371 ACD − 0.000009 BCD + 0.000002 ABCD(1)

where CapAds is the adsorption capacity (mg/g), A is the pH, B is the time (min), C is the temperature (°C), and D is the initial concentration (mg/L).

**Figure 6:**
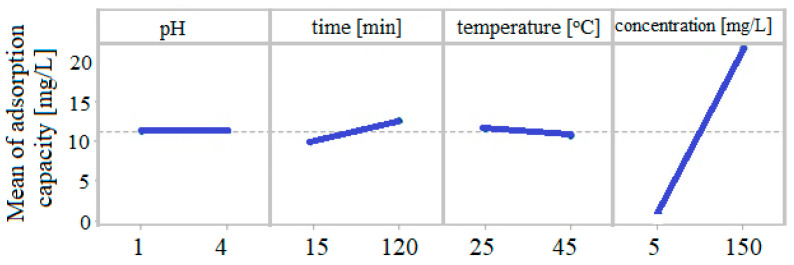
The main effects of the control factors on the adsorption capacity.

The Pareto diagram suggests that the initial concentration (D), the time (B), the interaction time–initial concentration (BD), and the interaction temperature–initial concentration (CD) have a significant influence on the adsorption capacity (q). The main effects of the control factors on the adsorption capacity are shown in Figure 6.

The pH and temperature do not influence the adsorption capacity, while the contact time has a slightly positive effect, and the initial concentration has a strong positive effect on the adsorption capacity.

**Figure 7:**
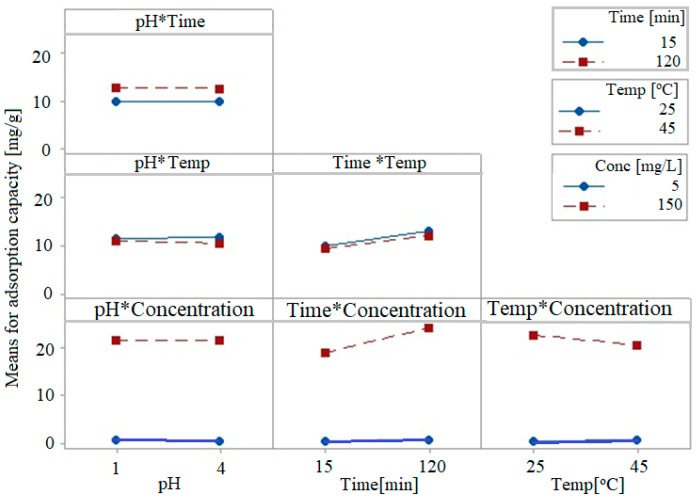
Interactions between the control factors and the response of the adsorption process (adsorption capacity).

The interplay between the control factors and the response of the adsorption process (adsorption capacity) is shown in Figure 7. The lack of parallelism confirms that the interactions between the considered control parameters are significant. Thus, the time–initial concentration interaction and the temperature–initial concentration interaction (to a lesser extent) significantly affect the adsorption capacity. These interactions can change the main effect of the controllable factors.

Note that these interactions can mask the control factors’ main effects.

**Figure 8:**
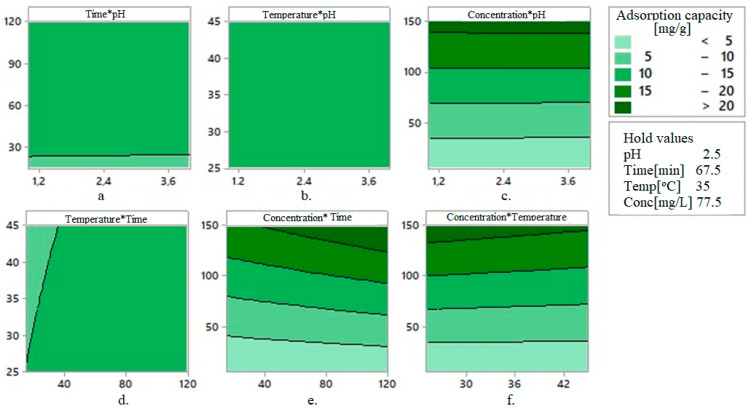
2D contour curves for the interaction (**a**) time×pH, (**b**) temperature×pH, (**c**) concentration×pH, (**d**) temperature×time, (**e**) concentration×time, and (**f**) concentration×temperature when the response is the adsorption capacity.

**Figure 9:**
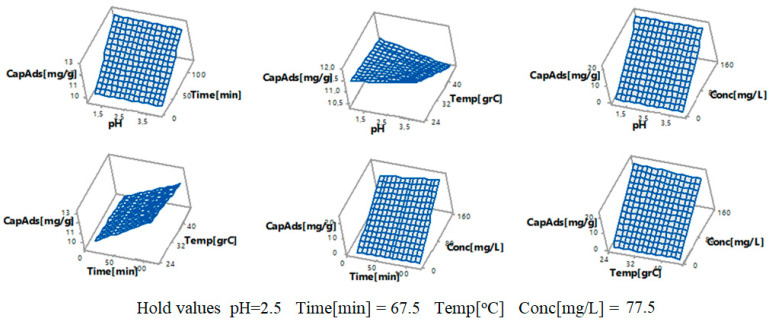
3D contour curves for the interactions time×pH, temperature×pH, concentration×pH, temperature×time, concentration×time, and concentration×temperature when the response is the adsorption capacity (CapAds (mg/g)).

Figure 8 and Figure 9 present the 2D contour curves and 3D contour curves of the interactions between the factors, considering that the response is the adsorption capacity. They show a linear correlation between the response and the control factors. In Figure 8, the more intense the colors are, the higher the correlation between the adsorption capacity and the factors are.

The results from Figure 8 are concordant with those from the experiments (pH = 2, contact time = 60 min, temperature = 35 °C, and concentration = 75 mg Au(III)/L) [50].

Figure 9 presents almost plane surfaces that show the dependence between the adsorption capacity, as the dependent variable, on pairs of factors (time and pH, temperature and pH, pH and initial gold concentration, time and temperature, initial gold concentration and time, and initial gold concentration and temperature) as the independent variables. Given the values of the two parameters, one can estimate the adsorption capacity.

**Figure 10:**
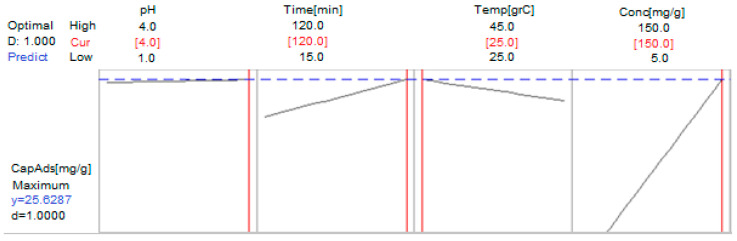
Optimization of the adsorption process of Au (III) on the Am-L-GA material.

Using MINITAB, we optimized the chemical process (the adsorption Au (III)) by establishing the most favorable control factors’ values for maximizing the adsorption capacity of the studied adsorbent material, Am-L-GA. The optimization results of the gold adsorption process on Am-L-GA are shown in Figure 10.

The maximum adsorption capacity (25.63 mg/g) is obtained at pH = 4, after 120 min, at a temperature of 25 °C, and with an initial gold concentration of 150 mg/L.

#### 3.2.2. Phase 2: Nonlinear Experiments—Response Surface Design (RSD) and Optimization of the Au (III) Adsorption Process on the Am-L-GA Material

Only two factors (the contact time and the initial concentration of Au (III)) have a significant effect on the studied adsorption process; in fact, on the adsorption capacity of the Am-L-GA material, a nonlinear response surface experiment is proposed, with the contact time values being between 110 and 140 min, and initial gold concentration being between 140 and 160 mg/L, at pH = 4 and temperature = 25 °C.

The response surface design was of the “Central Composite with Face Centered (CCFC)” type. Twelve runs were required to perform this model. The analysis of variance (ANOVA) in the CCFC design is presented in Table 2.

DF represents the degree of freedom, Adj SS is the adjusted squared sums, Adj MS is the adjusted mean squared (obtained by the ratio between Adj SS and DF), and F-value is the value of the Fisher test statistics. The determination coefficient R^2^ is 75.04%.

The ANOVA analysis shows that the nonlinear model is significant (*p*-value = 0.044 < 5%), so at least one term in the model has a significant impact on the average adsorption capacity.

The nonlinear effect (Square) (respectively, the second power terms) causes a curvature (nonlinearity) in the response (adsorption capacity). Since the *p*-value = 0.176 > 0.05, the nonlinear effect is not significant.

Neither Contact Time*Contact Time (*p*-value = 0.168) nor Initial Concentration*Initial concentration (*p*-value = 0.251) gave a significant response curvature (adsorption capacity).

The 2-way interactions are not significant (*p*-value = 0.077 > 0.05). Therefore, the linear effects are not influenced by the interactions, so they can be interpreted correctly.

The linear effect (*p* = 0.029, so *p* < 5%) is significant. As the interactions are not significant, the linear effect is not masked by the interactions. The significant linear effect is that of the initial concentration of Au (III) (*p* = 0.010 < 0.05).

From the experiment, it is observed that the contact time is significant, and the initial concentration has a strong and positive effect, offering a maximum Au (III) adsorption capacity for initial concentration values between 140 and 160 mg/L.

**Figure 11:**
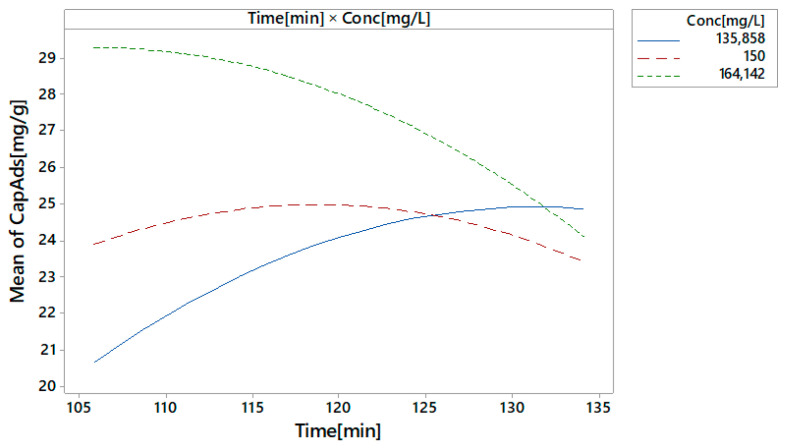
The contact time–initial concentration interactions.

The contact time–initial concentration interactions are shown in Figure 11.

The curves for the initial gold concentration of 150 and 164 mg/L are almost parallel and horizontal, which means that the contact time–initial concentration interaction does not have a significant effect. It is observed that there are slight interactions between the initial concentration of 136 mg/L and that of 150 mg/L. The interactions do not mask the main effects.

To validate the design, after removing the interactions Contact time (min) ×.*Contact time (min), Initial concentration (mg/L)*Initial concentration (mg/L), a residual analysis was performed. The Anderson–Darling test provides the associated statistics value, AD = 0.168, and *p*-value = 0.921. The statistics of the Levene test was 0.51, with a *p*-value = 0.488, whereas the correlogram proved that the residuals are not correlated. Therefore, the hypothesis that the residual is independently identically distributed cannot be rejected, so the design is correct from a statistical viewpoint.

**Figure 12:**
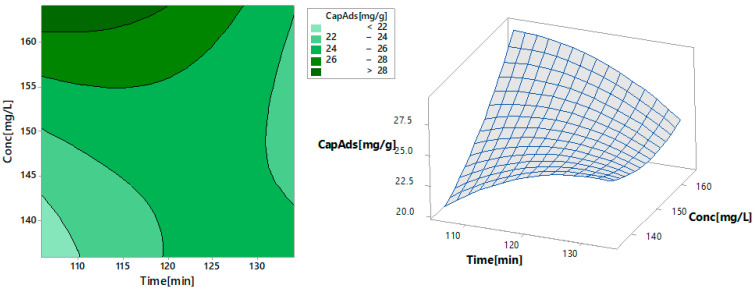
The contour (**left**) and surface (**right**) curves for the contact time and initial concentration if the response is the adsorption capacity.

Figure 12 shows the contour and surface curves for two of the control factors (contact time and initial concentration) when the response is the adsorption capacity. The adsorption capacity shows a nonlinear behavior. The left-hand side of the chart indicates—in dark green—the region with the highest adsorption capacity for a time lower than 127 min. The highest estimated value of the adsorption capacity was obtained for a maximum contact time of about 110 min and an Au (III) initial concentration of about 165 mg/L. At the opposite pole, the lowest adsorption capacity—represented by light-green—was obtained for contact times lower than 110 min and concentrations lower than 142 mg/L. Similar results are provided by the 3D representation of the adsorption capacity (on the *z*-axis) versus time and initial gold concentration (on the x and y axes) at the right-hand side of Figure 12.

**Figure 13:**
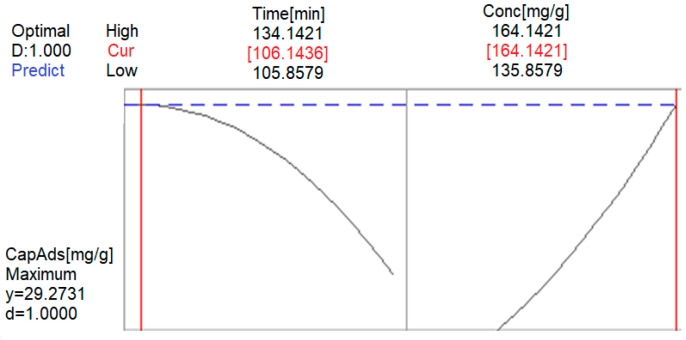
Optimization of the response adsorption process for Phase 2.

Using the software optimization facility, one can determine the optimal conditions for the contact time and the initial concentration, to obtain the maximum response for the adsorption capacity. This adsorption process response optimization is illustrated in Figure 13 and presented in Table 3.

Optimizing the adsorption process response for a contact time value of about 106 min and an initial Au (III) concentration of about 164 mg/L leads to an adsorption capacity between 22.45 mg/g and 28.95 mg/g.

Based on the regression equation for the Au (III) adsorption capacity, the maximum contact time position (MaxTime) can be analyzed as a function of the initial concentration. Equation (2) gives this dependence:

(2) MaxTime (conc)=−0.88771×conc+251.8967

where conc = initial concentration (mg/L), R^2^ = 0.92, and the standard deviations corresponding to the coefficients are 0.281 and 0.564.

To validate Model (2), statistical tests on the regression coefficients (*t*-tests) and the model on its whole (F-test) were performed. The corresponding *p*-values were less than 0.05, so the coefficients and the model are significant.

For the residual in Model (2), a normality analysis (Q-Q plot and Kolmogorov–Smirnov), homoscedasticity test, and autocorrelation test were performed.

**Figure 14:**
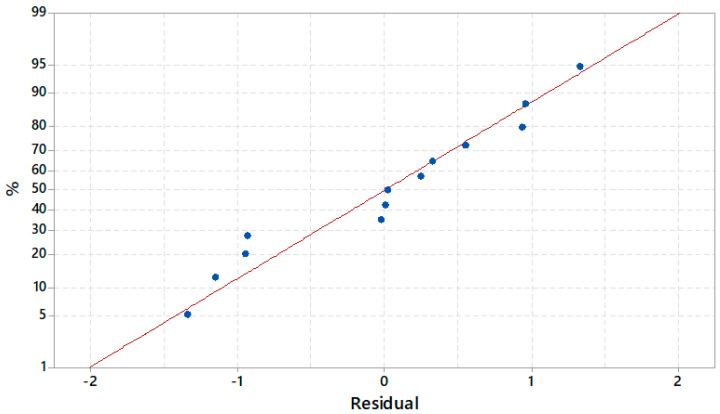
Q − Q plot of residual in Model (2).

The Kolmogorov–Smirnov test results could not reject the hypothesis that the residuals are Gaussian, confirming the Q-Q plot finding (Figure 14) that shows that the residual values (the blue dots) are close to the line representing the theoretical distribution.

The Levene test confirmed that the residuals have a constant variance, whereas the correlogram indicated the absence of autocorrelation.

Considering the above results, one may conclude that Model (2) is correct from a statistical viewpoint.

**Figure 15:**
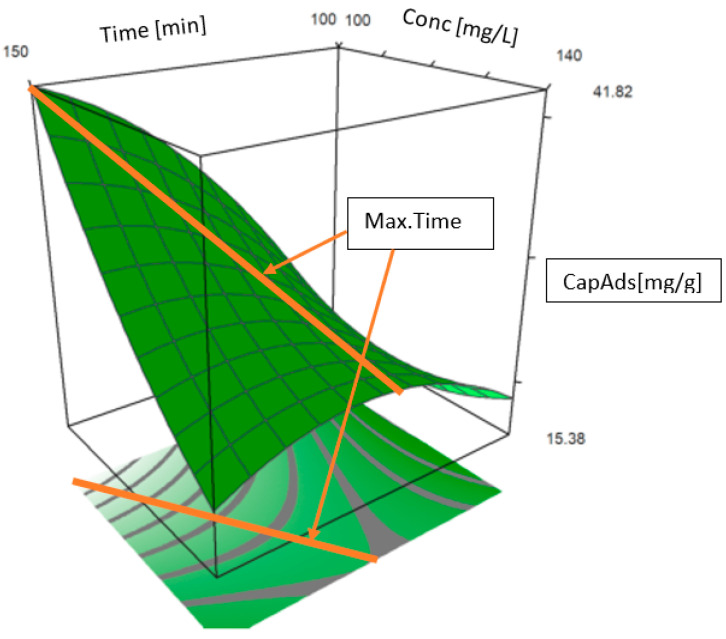
Dependence of the contact time upon the initial Au (III) concentration during the adsorption process using Am-L-GA.

The dependence of the contact on the initial Au (III) concentration during the adsorption process using Am-L-GA is represented in Figure 15. For example, considering an initial concentration of 100 mg/L, the maximum contact time is 163.126 min, and for an initial concentration of 140 mg/L, the maximum contact time should be 127.618 min.

The studies [37,38] show a good concordance between the extraction process carried under the statistically optimized conditions and the theoretical results. A difference of less than 5% was reported by Mendez and Martins [36]. Oluwabunmi [38] found an increase of 87.13% in the recovery. Teimouri et al. [37] performed gold extraction under optimum experimental conditions for 24 h and found 35.7% gold extraction, consistent with the model, which predicted 35.2%. Our study confirms the efficiency of the proposed optimization method since the theoretical results obtained in Phase I are concordant with the experimental ones (as mentioned above). Still, the predictions obtained in Phase II should be further confirmed.

After the gold adsorption onto the obtained adsorbent material, it is possible to recover the metal by performing calcination of the impregnated material at a temperature of 873 K for a minimum of 240 min. Subsequently, the metallic gold is recovered directly from the obtained ash. Another procedure is presented in [44], which uses HNO_3_ (5%) as the desorption agent, in five cycles.

## 4. Conclusions

Am-L-GA is a new material obtained from a commercial Amberlite XAD7-type resin functionalized by impregnation with L-glutamic acid. Previous studies [44,50] emphasized a higher adsorption capacity for Au(III) of this material compared to Amberlite XAD 2000—12.3 mg/g [57]; Dowex M 4195—8.1 mg/g [58]; sulphuric acid cross-linked alginate powder—5.64 mg/g [59]; thiourea modified alginate powder—6.40 mg/g [60]; porous epichlorohydrin/thiourea modified alginate (PETA)—1.97 mg/g [61]; and Amberlite XAD 7—1 mg/g [50,62]. Therefore, this study aimed to optimize Au(III)’s recovery process from industrial cyanide solutions, increasing the extracted gold quantity, and selecting the optimal conditions for getting the best adsorption capacity in industrial processes of gold recovery from waste solutions.

Since between the material morphology and its adsorption capacity there is a strong relationship [53,54,55,56], the first step was the material characterization. Formations with ≈0.45 μm and ≈1.70 μm heights have been detected, resulting in high skewness and kurtosis of the surface roughness. These characteristics, together with the low porosity (indicated by the Ssk values), supports why Am-L-GA is recommended as a material with good adsorption capacity [44,50,53,54,55,56].

A factorial design was used for optimizing similar processes with good results, our study confirming these previous findings [50,57,62,63,64,65]. The Au (III) adsorption process depends essentially on the contact time and the initial concentration. The results from Phase I show good concordance with those from [50]; the adsorption capacity was estimated with 98.7% accuracy. The maximum adsorption capacity in this phase was determined to be 25.63 mg/g (compared with 26 mg/g in the experiments), obtained at pH = 4, after 120 min, at a temperature of 25 °C, and an initial gold concentration of 150 mg/L. The results from Phase II indicate a linear dependence between the contact time and the initial gold concentration. Extending the domain of the experimental research, by optimizing the adsorption process, it was found that for a contact time ~106 min and an initial Au (III) concentration of ~164 mg/L, the adsorption capacity values range between 22.45 mg/g and 28.95 mg/g, at a 95% confidence level.

This study recommends Am-L-GA as a new material with superior adsorption capacity for gold retrieval from industrial solutions and confirms the ability of the factorial design for optimizing such process, cross-validating previous research [41,42,43,57,58,59,60,61,62,63,64,65].

## Figures and Tables

**Table 1:** Roughness parameters.

Sample Ame	Area(µm^2^)	Sa(µm)	Sq(µm)	Sp(µm)	Sv(µm)	Sy(µm)	Sku	Ssk
Am-L-GA	677.94	0.108	0.238	1.825	−0.101	1.926	28.832	4.925

**Table 2:** ANOVA results in the CCFC design.

Source	DF	Adj SS	Adj MS	F	*p*-Value
Model	5	26.8843	5.3769	4.21	0.044
Linear	2	15.6554	7.8277	6.13	0.029
Contact time (min)	1	0.2283	0.2283	0.18	0.045
Initial concentration (mg/L)	1	15.4270	15.4270	12.08	0.010
Square	2	5.7578	2.8789	2.25	0.176
Contact time (min)*Contact time (min)	1	3.0209	3.0209	2.37	0.168
Initial concentration (mg/L) * Initial concentration (mg/L)	1	1.9980	1.9980	1.56	0.251
2-way interaction	1	5.4711	5.4711	7.28	0.077
Contact time (min) * Initial concentration (mg/L)	1	5.4711	5.4711	7.28	0.077
Error	7	8.9405	1.2772		
Lack of Fit	3	7.1395	2.3798	5.29	0.071
Pure Error	4	1.8010	0.4502		
Total	12	35.8248			

**Table 3:** Optimization of the response of the Au (III) adsorption process for Phase 2.

Parameters	Minim	Target
Adsorption capacity (mg/g)	22.4457	28.9523
**Global solution**		
Contact time, (min)	106.14	
Initial concentration (mg/L)	164.142	
**Answer predict**		
Adsorption capacity (mg/g)	29.2731	

## Data Availability

Date are available on request.
